# TNF-α and CD8^+^ T Cells Mediate the Beneficial Effects of Nitric Oxide Synthase-2 Deficiency in Pulmonary Paracoccidioidomycosis

**DOI:** 10.1371/journal.pntd.0002325

**Published:** 2013-08-01

**Authors:** Simone Bernardino, Adriana Pina, Maíra Felonato, Tânia A. Costa, Eliseu Frank de Araújo, Cláudia Feriotti, Silvia Boschi Bazan, Alexandre C. Keller, Katia R. M. Leite, Vera L. G. Calich

**Affiliations:** 1 Departamento de Imunologia, Instituto de Ciências Biomédicas, Universidade de São Paulo, São Paulo, Brasil; 2 Departamento de Microbiologia, Imunologia e Parasitologia, Universidade Federal de São Paulo, São Paulo, Brasil; 3 Departamento de Patologia, Hospital Sírio Libanês de São Paulo, São Paulo, Brasil; University of California San Diego School of Medicine, United States of America

## Abstract

**Background:**

Nitric oxide (NO), a key antimicrobial molecule, was previously shown to exert a dual role in paracoccidioidomycosis, an endemic fungal infection in Latin America. In the intravenous and peritoneal models of infection, NO production was associated with efficient fungal clearance but also with non-organized granulomatous lesions. Because paracoccidioidomycosis is a pulmonary infection, we aimed to characterize the role of NO in a pulmonary model of infection.

**Methodology/Principal Findings:**

C57Bl/6 wild type (WT) and iNOS^−/−^ mice were i.t. infected with 1×10^6^ *Paracoccidioides brasiliensis* yeasts and studied at several post-infection periods. Unexpectedly, at week 2 of infection, iNOS^−/−^ mice showed decreased pulmonary fungal burdens associated with an M2-like macrophage profile, which expressed high levels of TGF-β impaired ability of ingesting fungal cells. This early decreased fungal loads were concomitant with increased DTH reactions, enhanced TNF-α synthesis and intense migration of activated macrophages, CD4^+^ and CD8^+^ T cells into the lungs. By week 10, iNOS^−/−^ mice showed increased fungal burdens circumscribed, however, by compact granulomas containing elevated numbers of activated CD4^+^ T cells. Importantly, the enhanced immunological reactivity of iNOS^−/−^ mice resulted in decreased mortality rates. In both mouse strains, depletion of TNF-α led to non-organized lesions and excessive influx of inflammatory cells into the lungs, but only the iNOS^−/−^ mice showed increased mortality rates. In addition, depletion of CD8^+^ cells abolished the increased migration of inflammatory cells and decreased the number of TNF-α and IFN-γ CD4^+^ and CD8^+^ T cells into the lungs of iNOS^−/−^ mice.

**Conclusions/Significance:**

Our study demonstrated that NO plays a deleterious role in pulmonary paracoccidioidomycosis due to its suppressive action on TNF-α production, T cell immunity and organization of lesions resulting in precocious mortality of mice. It was also revealed that uncontrolled fungal growth can be overcome by an efficient immune response.

## Author Summary

Paracoccidiodomycosis is a human systemic mycosis endemic in Latin America that has a wide spectrum of manifestations ranging from localized to fatal disseminated forms. Both in humans and experimental models, immunoprotection is mediated by T cell immunity whereas immunosuppression is associated with the severe forms of the disease. The literature shows that nitric oxide (NO) produced by the enzyme nitric oxide synthase-2 (NOS2 or iNOS) is the major fungicidal component of phagocytic cells. The role of NO production was previously investigated in the intra-peritoneal and intravenous murine models of *Paracoccidioides brasiliensis* infection. The human paracoccidioidomycosis is believed to be acquired by the respiratory route, thus our study aimed to characterize the role of NO production in a pulmonary model of infection. We verified that, paradoxically, absence of NO production by iNOS- deficient mice resulted in less severe disease and increased survival times. This was associated with increased development of cellular immunity and enhanced synthesis of TNF-α which enhances cell migration to the site of infection and contributes to the better organization of lesions. Our work highlighted the deleterious effect of excessive NO production in pulmonary paracoccidioidomycosis, and demonstrated that uncontrolled fungal growth can be overridden by an efficient immune response.

## Introduction

Phagocytes are important effector cells of innate and adaptative immunity and use several mediators and mechanisms to control pathogen growth. The production of nitric oxide (NO) has been shown to be an important microbicidal mechanism of macrophages in the protective immune responses against different pathogens [1], [2], [3], [4]. NO is generated from the amino acid L-arginine by the catalytic action of the inducible isoform of NO synthase (iNOS or NOS2) [5]. The synergistic interaction of NO with hydrogen peroxide (H_2_O_2_) or superoxide (O_2_^−^) anion can generate extremely potent oxidizing compounds resulting in cell damage and microbicidal activity [6], [7]. Besides its action on pathogens viability, there are also evidences that NO has an inhibitory effect in the innate and adaptive immunity of hosts. For example, it reduces the antigen-presenting ability of pulmonary dendritic cells, inhibits MHC class II antigen expression, controls the production of cytokines and expression of costimulatory and adhesion molecules [8], [9]. The iNOS gene expression is regulated by an ever-increasing number of agonists, especially proinflammatory cytokines such as IFN-γ and TNF-α and bacterial products such as lipopolysaccharides from Gram-negative bacteria [10], [11]. On the other hand, type 2 cytokines, especially IL-4, IL-10 and TGF-β, were shown to inhibit NO production [12], [13], [14].

Similarly with other microorganisms, the production of NO has been associated with the protective immunity against several fungal pathogens [15], [16]. Recent evidences, however, suggested that certain fungal species such as *Cryptococcus neoformans* and *Aspergillus fumigatus* developed ingenious mechanisms to evade nitric oxide-dependent death [17], [18].

Paracoccidioidomycosis (PCM), a fungal disease caused by the inhalation of *P. brasiliensis* spores, presents a wide spectrum of immunopathological manifestations [19]. Patients with benign PCM usually develop adequate cellular immune responses and their antigen-stimulated leukocytes preferentially secrete type 1 cytokines; in contrast, patients with the severe form of the disease show impaired cell mediated immunity and type 2-skewed immune response [20], [21], [22]. Recent investigations, however, indicate that other regulatory mechanisms, not involving Th1/Th2 cells, play an important role in the immunopathogenesis of PCM [21].

Although the mechanisms involved in resistance to *P. brasiliensis* infection are not completely understood, it appears that alveolar macrophages have a fundamental role, acting as the first line of host defense. The enhanced fungicidal ability of cytokine-activated macrophages was shown to be mainly mediated by NO [23], [2]. Despite this protective activity, in some studies NO production was associated with suppression of lymphoproliferation and MHC class II expression [24], [25]. Interestingly, in *P. brasiliensis* infection we could detect an inverse correlation between TNF-α synthesis and NO production. Peritoneal and alveolar macrophages from resistant A/J mice in vitro infected with *P. brasiliensis* yeasts secreted high TNF-α levels, low NO amounts and displayed low fungicidal ability associated with enhanced TGF-β secretion. In contrast, macrophages from susceptible B10.A mice secreted high NO levels, presented efficient fungal killing but produced low levels of TNF-α [26], [27]. NO deficiency was also associated with organized granulomas of i.p. infected mice [28], while exacerbated inflammatory reactions and cytokines production was described in i.v. infected mice [29].

Because *P. brasiliensis* infection is acquired by the respiratory route and the role of NO was never investigated in the pulmonary model of PCM, we aimed to further understand the immunoregulatory function of this mediator using i.t. infected iNOS-deficient (iNOS^−/−^) and normal (WT) C57BL/6 mice. We could characterize the temporal effects of NO synthesis in the control of fungal growth. At week 2 of infection, absence of NO results in lower fungal loads but at week 10, increased numbers of yeasts were detected in the lungs of iNOS^−/−^ mice. Unexpectedly, the deficient mouse strain showed increased survival times and this behavior was associated with high levels of TNF-α production increased and persistent delayed type hypersensitivity reactions and enhanced migration of activated T cells and macrophages into the lungs of infected mice. In addition, the increased fungal loads lately developed by iNOS-deficient mice appeared to be contained by better organized granulomatous lesions. Furthermore, in vivo depletion experiments showed that the protective effect of iNOS deficiency was mainly mediated TNF-α and the expansion of IFN-γ and TNF-α CD4^+^ and CD8^+^ T cells.

## Materials and Methods

### Ethics statement

Animal experiments were performed in strict accordance with the Brazilian Federal Law 11,794 establishing procedures for the scientific use of animals, and the State Law establishing the Animal Protection Code of the State of São Paulo. All efforts were made to minimize suffering, and all animal procedures were approved by the Ethics Committee on Animal Experiments of the Institute of Biomedical Sciences of University of São Paulo (Proc.76/04/CEEA).

### Mouse strains

Breeding pairs of homozygous iNOS-deficient (iNOS^−/−^) and wild type (WT) control C57BL/6 mice (intermediate susceptibility to *P. brasiliensis*) were bred at the University of São Paulo animal facilities under specific-pathogen-free (SPF) conditions in closed-top cages. Clean food and water were given ad libitum. Mice were 8 to 11 weeks of age at the time of infection, and procedures involving animals and their care were approved by the Ethics Committee on Animal Experiments of our Institution.

### Fungus and mice infection

The *P. brasiliensis* 18 isolate, which is highly virulent, was used throughout the study. To ensure the maintenance of its virulence, the isolate was used after three serial animal passages [30]. *P. brasiliensis* 18 yeast cells were then maintained by weekly subcultivation in semisolid Fava Netto culture medium [31] at 35°C and used on the seventh day of culture. The fungal cells were washed in phosphate-buffered saline (PBS; pH 7.2), counted in a hemocytometer and the concentration was adjusted to 20×10^6^ fungal cells ml^−1^. The viability of fungal suspensions, determined by Janus Green B vital dye (Merck, Darmstadt, Germany) [32], was always higher than 80%. Mice were anesthetized and submitted to i.t. *P. brasiliensis* infection as previously described [33]. Briefly after intraperitoneal anesthesia, the animals were infected with 1×10^6^ *P. brasiliensis* 18 yeast cells, contained in 50 µL of PBS, by surgical i.t. inoculation, which allowed dispensing of the fungal cells directly into the lungs. The skins of the animals were then sutured, and the mice were allowed to recover under a heat lamp. Mice were studied at several time points after infection.

### Assay for organ colony forming units (CFU)

The number of viable microorganisms in infected organs (lung, liver and spleen) from experimental and control mice were determined by counting the number of CFU. Animals (n = 6–8) from each group were sacrificed, and the enumeration of viable organisms was done as previously described [34]. Briefly, aliquots (100 µL) of the cellular suspensions and serial dilutions were plated on brain heart infusion agar (Difco, Detroit, USA) supplemented with 4% (vol/vol) horse serum (Instituto Butantan, São Paulo, Brazil) and 5% *P. brasiliensis* 192 culture filtrate, the latter constituting a source of growth-promoting factor. The plates were incubated at 35°C, and colonies were counted daily until no increase in counts was observed. The number (log_10_) of viable *P. brasiliensis* colonies per gram of tissue were expressed as means ± standard errors (SEs).

### Measurement of cytokines

Mice (n = 6–8) were infected i.t. with *P. brasiliensis*, their right lung and liver of anti-TNF-α and IgG treated mice, were removed aseptically and individually disrupted in 5.0 mL of PBS. Supernatants were separated from cell debris by centrifugation at 2,000× g for 15 min, passed through 0.22 µm pore-size filters (Millipore, Bedford, Mass, USA), and stored at −70°C. The levels of IL-2, IL-12, IFN-γ, TNF-α, IL-4, IL-5, IL-10 and TGF-β were measured by capture enzyme-linked immunosorbent assay (ELISA) with antibodies pairs purchased from Pharmingen (Pharmingen, San Diego, CA, USA). The ELISA procedure was performed according to the manufacture's protocol. The concentrations of cytokines were determined with reference to a standard curve for several twofold dilutions of murine recombinant cytokines. As an addition control, lung homogenates were added to recombinant cytokines used to obtain standard curves; no interference was detected, indicating the absence of inhibitory substances (e.g., soluble cytokine receptors).

### Studies with alveolar macrophages

Two and ten weeks after i.t. infection, lungs of mice were lavaged by repeated injections of 0.5 ml of sterile PBS (final volume 2.0 ml) after cannulation of the trachea with polyethylene tubing which was attached on a tuberculin syringe. An aliquot of the recovered bronchoalveolar lavage fluid (BALF) was assayed by CFU to determine the presence of viable yeasts. Then, the remaining BALFs obtained from individual mice were spun at 1200 rpm, the supernatants removed and alveolar macrophages cultivated to characterize the presence of viable fungal cells. Cell pellets were resuspended in RPMI containing 10% fetal calf serum, 2 mM L-glutamine, 100 U/ml penicillin and 100 µg/ml streptomycin, adjusted at 4×10^5^/ml of culture medium and 0.5 ml dispensed in 24-well tissue culture plates for a 2 h adhesion step. Non-adherent cells were discarded and some cultures treated with 0.5 ml of culture medium supplemented with 100 U/ml of IFN-γ (Pharmingen, San Diego, CA, USA) and cultivated for 48 h. Plates were then centrifuged, supernatants stored, cells disrupted by five washes with 0.5 ml of distilled water and suspensions collected in individual tubes. Pellets were resuspended in culture medium, and aliquots (100 µL) and their serial dilution were assayed for the presence of viable yeasts. In addition, NO and H_2_O_2_ levels were determined in the supernatants of alveolar macrophages cultures.

### Fungicidal ability of peritoneal macrophages

Peritoneal macrophages from WT and iNOS ^−/−^ C57BL/6 mice were induced by i.p. injection of brewer thioglycollate medium (Difco, Detroit, MI, USA). Macrophages were isolated by adherence (2 h at 37°C in 5% CO_2_) to plastic-bottom tissue-culture plates (1×10^6^ cells/well in 24 well plates), cultivated overnight with fresh complete medium in the presence or absence of recombinant IFN-γ (40 ng/ml, BD-Pharmingen San Diego, CA, USA), TNF-α (20 ng/ml, BD-Pharmingen San Diego, CA, USA) or 1-methyl-DL-tryptophan (1 MT, 1 mM in culture medium, Sigma Aldrich, St. Louis, MO, USA), a specific inhibitor of 2,3 indoleamine dioxygenase. Macrophage cultures were infected with *P. brasiliensis* yeasts in a macrophage∶yeast ratio of 12.5∶1. After 48 h of culture, plates were centrifuged and supernatants removed. The wells were washed with distilled water to lyse macrophages, the suspensions collected and assayed for the presence of viable yeasts. All assays were done with five wells per condition in over three independent experiments.

### Phagocytic assays using FITC-labeled *P. brasiliensis*

For phagocytic assays, macrophages from WT and iNOS ^−/−^ mice were infected with heat-inactivated, FITC labeled *P. brasiliensis* yeasts at a macrophage∶yeast ratio of 1∶1 for 2 h at 37°C in 5% CO_2_ to allow fungi adhesion and ingestion as previously described [35]. Some macrophage cultures were treated with IFN-γ (40 ng/ml, BD-Pharmingen), or TNF-α (20 ng/ml BD-Pharmingen) overnight, before infection. Macrophages were gently washed twice with PBS and cells detached from plastic with fresh cold medium and a rubber cell scraper on ice. The cells were transferred to tubes, centrifuged (400×g. 10 min., 4°C), and the pellets were labeled with anti-F4/80 (APC) antibodies (eBioscience, San Diego, CA, USA). The cells were washed twice in PBS, the pellets were suspended in 200 µL of PBS 1% FCS and were immediately read on FACScalibur (Becton Dickinson, Franklin Lakes, NJ, USA) and data analyzed using the FlowJo software program (Tree Star, Ashland, OR, USA).

### Quenching assay

For the distinction between internalized and surface-bound yeasts (FITC- labeled *P.brasiliensis* particles), trypan blue (TB, 250 µg/mL, Sigma Aldrich, St. Louis, MO, USA) was used for quenching the green surface-bound fluorescence on macrophages. TB quenching technique was performed as described by Busetto et al. [36] with minor modifications. Phagocytic assays were performed as above described and adherent/ingested cells measured using the FL1 and FL4 channels of a FACscalibur cytometer. Cell suspensions were then treated in an ice bath with 0.1 ml of a TB solution prepared in 0.1 M citrate buffer, pH 4.0, lowering samples pH to nearly 4.0, thereby optimizing the TB quenching effect. After 1 min of incubation in ice bath, the samples were again analyzed. APC-labeled macrophages were gated, and FL1 and FL3 channels used to discriminate ingested (green fluorescent, FL1) from adherent (red fluorescent, FL3) yeasts.

### Quantitative real-time PCR

Total RNA was extracted from cultures of normal or *P. brasiliensis*-infected macrophages using the TRIzol reagent (Invitrogen, Carlsbad, CA, USA) according to the manufacturer's instructions. The RNA concentrations were determined by spectrophotometer readings at an absorbance of 260 nm. First-strand cDNAs were synthesized from 2 µg RNA using the High Capacity RNA-to-cDNA kit (Applied Biosystems, Foster City, CA, USA) according to the manufacturer's instructions. Real-time polymerase chain reaction (RT-PCR) was performed using the TaqMan real-time PCR assay (Applied Biosystems) for the following molecules: ARG1 (Mm00475988_m1), NOS2 (Mm00440502_m1), IL-12 p40 (Mm00434174_m1), TGF-β (Mm00441727_m1), TNF-α (Mm99999068_m1), IDO (Mm00492586_m1). Cycling conditions were as follows: 10 min at 95°C, followed by 45 cycles of 20 s at 95°C, 20 s at 58°C, and 20 s at 72°C. Analysis was performed with the ABI PRISM 7000 sequence detection system (Applied Biosystems). GAPDH was used as an internal control. All values were normalized to GAPDH, and the relative gene expression was calculated using the Pfaffl method [37]

### Nitric oxide production

Nitric oxide production was quantified by the accumulation of nitrite in the supernatants by a standard Griess reaction. Briefly, 50 µL of supernatants was removed from 24-well plates and incubated with an equal volume of Griess reagent (1% sulfanilamide, 0,1% naphthylene diamine dihydrochloride, 2,5% H_3_PO_4_) at room temperature for 10 min. The absorbance at 550 nm was determined with a microplate reader. The conversion of absorbance to micromolar NO was deduced from a standard curve by using a known concentration of NaNO_2_ diluted in RPMI medium [38]. All determinations were performed in triplicates and expressed as micromolar NO.

### DTH assay

DTH reactions were evaluated employing a footpad test previously described [34]. Briefly, mice were inoculated with 25 µl of a soluble *P. brasiliensis* antigen [31] and footpad thickness was measured with a caliper (Mitutoyo, Tokyo, Japan) immediately before and 24 h after antigen inoculation. The increase in thickness was calculated and expressed in millimeters. Non-infected mice submitted to the footpad test were used as controls.

### Lung leukocytes isolation

Lungs from each mouse were excised, washed in PBS, minced, and digested enzymatically for 1 hour in 15 mL/lung of digestion buffer [RPMI, 5% fetal calf serum, 1 mg/mL collagenase and 30 µg/mL DNase (Sigma Aldrich, USA)]. After erythrocyte lysis using NH_4_Cl buffer, cells were washed, resuspended in complete media, and centrifuged for 30 minutes at 2,000× *g* in presence of 20% Percoll (Sigma) to separate leukocytes from cell debris and epithelial cells. Total lung leukocyte numbers were assessed in the presence of trypan blue using a hemocytometer; viability was always higher than 85%. The absolute number of a leukocyte subset was equal to the percentage of that cell subset multiplied by the total number of leukocytes recovered from the digested lung/100.

### Flow cytometry analysis

For surface staining alone, leukocytes were washed and resuspended at a concentration of 1×10^6^ cells/mL in staining buffer (PBS 1×, 2% serum calf bovine and 0,5% NaN_3_). Fc receptors were blocked by the addition of unlabeled anti-CD16/32 (Fc block; BD Pharmingen, San Diego, CA, USA). The leukocytes were then stained for 20 min at 4°C with the optimal dilution of each antibody. Anti-CD4, CD8, CD69, CD25, CD40 CD80, CD86, CD11b and CD11c FITC or PE-conjugated antibodies were from BD Pharmingen. Cells were washed twice with staining buffer resuspended in 100 µl, and an equal volume of 2% formalin was added to fix the cells. The stained cells were analyzed immediately on a FACScalibur equipment using the Cell-Quest software (Becton & Dickinson, Sparks, MD, USA) gating on macrophages or lymphocytes as judged from forward and side light scatter. Ten thousands cells were counted and the data expressed as the percentage or the absolute number of positive cells which was calculated trough the percentage obtained by FACS and the number of cells determined in Neubauer chambers. The intracellular detection of FoxP3, the X-linked forkhead/winged helix transcription factor, in leukocytes obtained from the lung lesions was performed in fixed and permeabilized cells using Cytofix/Cytoperm (BD Biosciences, San Diego, CA, USA). Initially, the cells were labeled with antibodies for cell surface molecules such as FITC-conjugated anti-CD4 and PE-conjugated anti-CD25. Next, the cells were fixed, permeabilized, and stained with Cy-conjugated anti-FoxP3, for 1,5 h at 4°C. Cells were then washed twice with staining buffer, resuspended in 100 µl, and an equal volume of 2% formalin was added to fix the cells. A minimum of 20,000 events was acquired on FACScalibur flow cytometer (BD Pharmingen) using the Cell-Quest software (BD Pharmingen). The graphs represent the number of Foxp3^+^ cells in the gate of CD4^+^ CD25^+^ T cells. For intracellular cytokine (IL-4, IFN-γ and TNF-α) staining, cells were stimulated for 6 h in complete medium in the presence of 50 ng/ml phorbol 12-myristate 13-acetate, 500 ng/ml ionomycin (both from Sigma-Aldrich) and monensin (3 mM, eBioscience). After surface staining for CD4 (Pacific Blue anti-CD4) and CD8 (Alexa Fluor 488 anti CD8), cells were fixed, permeabilized, and stained by PerCP- Cy5.5 anti-IFN-γ, Pe-Cy7 anti-IL-4 and PE anti-TNF-α antibodies (eBioscience, San Diego,CA, USA). The cell surface expression of leukocyte markers as well as intracellular expression of IL-4, IFN-γ and TNF-α in lung infiltrating leucocytes, were analyzed in a FACScalibur flow cytometer (BD Pharmingen) using the FlowJo software (Tree Star, Ashland, OR, USA).

### Isolation of pulmonary dendritic cells (DCs)

In selected experiments, lungs from infected iNOS^−/−^ and WT mice were removed and digested enzymatically as above described. DCs were purified by magnetic cell sorting with microbeads (Miltenyi, Bergisch Gladbach, Germany) conjugated to hamster anti-mouse CD11c monoclonal antibodies. Positively selected DCs contained more than 90% CD11c^+^ cells. Cell-surface markers of pulmonary DCs were characterized by flow cytometry using monoclonal antibodies anti-CD11c PE-Cy7, anti-CD11b PerCP-Cy5.5, anti-CD8a-Alexa Fluor 488, and anti- B220-PE.

### In vivo neutralization of TNF-α activity and depletion of CD8^+^ cells

*P. brasiliensis*-infected (iNOS^−/−^ and WT) mice were given i.p. injections of 0.25 mg/0.5 mL of an anti-TNF-α MAb (MP6 XT 22.11), a rat IgG1 monoclonal antibody, against murine TNF-α [39], 4 h before the infection, and at days 6 and 12 postinfection. Normal rat IgG was given i.p. to mice as a control for antibody administration. Treated and untreated mice (n = 6–7) were studied at weeks 2 and 8 weeks after infection and mortality rates were also evaluated. H-35 (rat IgG1 anti-CD8α) hybridoma was grown in BALB/c nu/nu mice. Monoclonal antibodies (MAbs) were purified from ascites as previously described [40] and assayed for purity by sodium dodecyl sulfate-polyacrylamide gel electrophoresis. Groups (n = 6–7) of WT and iNOS^−/−^ mice were given 200 µg of anti-CD8α mAbs or normal rat IgG (controls) by the i.p. route, 48 and 24 h before infection and 150 µg of the mAbs or rat IgG at days 6 and 12 postinfection. The severity of infection and lung infiltrating leukocytes were characterized at week 2 after infection.

### Histopathologic analysis

Groups of iNOS^−/−^ mice and their normal counterparts were killed at the second, eighth and tenth week postinfection. Lungs were collected, fixed in 10% formalin and embedded in paraffin. Histolopathogical studies were also performed with anti-TNF-treated and untreated mice at week 8 after infection. Five-micrometer sections were stained by the hematoxilin-eosin (H&E) for an analysis of the lesions. Pathological changes were analyzed based on the size, morphology and cell composition of granulomatous lesions, presence of fungi and intensity of the inflammatory infiltrates.

### Mortality studies

Mortality studies were done with groups (n = 6–8) of iNOS^−/−^ and WT control mice inoculated i.t. with 1×10^6^ yeast cells or PBS. Deaths were registered daily for a 350-day period, and the median survival time postinfection was calculated. Mortality of untreated and anti-TNF-α -treated mice (n = 6–7) of both mouse strains were also studied. Experiments were repeated three times.

### Statistical analysis

All values are means ± SEM, unless otherwise indicated. Depending on the number of experimental groups, data were analyzed by Student's *t* test or two-way analysis of variance and the Bonferroni posttests to compare groups. Differences between survival times were determined with the LogRank test using GraphPad Prism software (GraphPad Software, San Diego, CA, USA). *P* value<0.05 was considered significant.

## Results

### Effect of iNOS deficiency in the severity of *P. brasiliensis* infection

**Figure 1:**
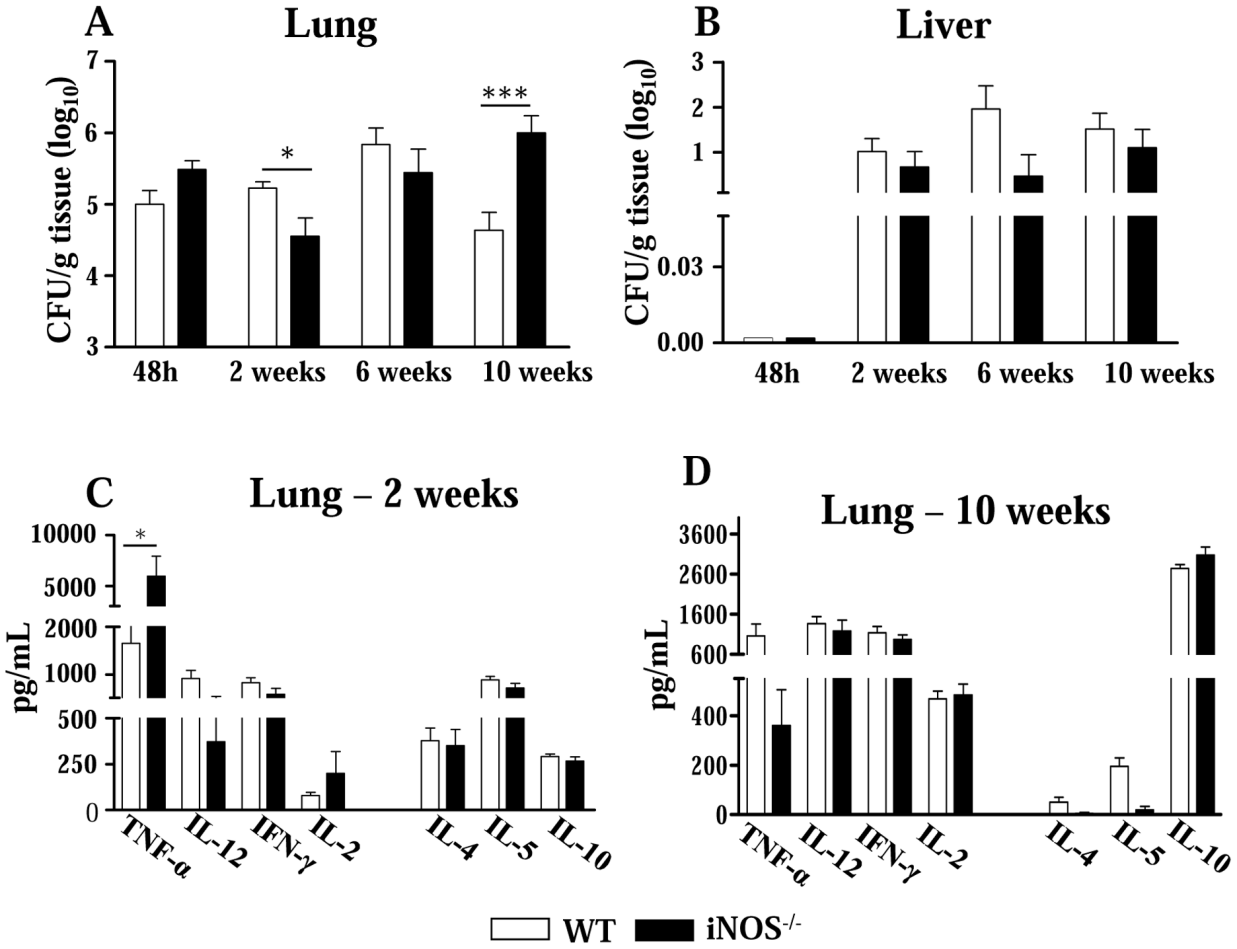
In pulmonary paracoccidioidomycosis, iNOS-deficiency alters fungal loads and synthesis of cytokines. (A, B) Recovery of CFUs from the lungs and liver of iNOS^−/−^ and WT control mice infected i.t. with 1×10^6^ yeasts. Early in infection (week 2), the lack of NO production results in lower fungal loads but at a late period (week 10), an increased pulmonary fungal burden was observed. The bars represent means ± SEM of log_10_ CFU obtained from groups of six to eight mice. The results are representative of three experiments with equivalent results. (C, D) Levels of type 1 and type 2 cytokines in lungs homogenates of iNOS^−/−^ and WT mice (n = 6–8). At the 2^nd^ (C) and 10^th^ (D) weeks after i.t. infection, lungs from iNOS^−/−^ and WT mice were collected and disrupted in 5.0 mL of PBS and supernatants were analyzed for cytokines content by capture ELISA. The bars depict means ± SEM of cytokine levels (6–8 animals per group). * (*P*<0.05) and *** (*P*<0.001) compared with WT controls.

The evolution of the disease of i.t. infected iNOS^−/−^ mice and their WT controls was monitored by CFU counts in the lung and liver at different postinfection periods (48 h, 2, 6 and 10 weeks) (Figure 1A and 1B). At the first 48 h of infection, an equivalent number of viable yeasts cells was recovered from lungs of both mouse strains. Interestingly, at the 2^nd^ week, iNOS deficiency resulted in decreased CFU counts in the lungs (4.55±0.86 log_10_ CFU/g of tissue), compared with normal controls (5.23±0.29 log_10_ CFU/g of tissue). No differences were noted in the dissemination to liver (Figure 1B) and spleen (data not shown). Although at the 6^th^ week both groups of mice showed equivalent pulmonary fungal loads, at week 10 postinfection the iNOS^−/−^ mice presented augmented fungal burden in the lungs (5.99±0.79 log_10_ CFU/g of tissue) relative to WT mice (4.64±0.80 log_10_ CFU/g of tissue). Again, no differences in fungal dissemination were observed. These data clearly showed the opposite temporal effect of NO: early in the infection, the absence of nitric oxide synthesis resulted in a protective effect, but at the chronic phase led to a more severe disease (Figure 1A).

Because the differences in pulmonary fungal burdens of iNOS^−/−^ and WT mice were detected at the 2^nd^ and the 10^th^ weeks postinfection, these periods were chosen to next determine the levels of type 1 (IFN-γ, TNF-α, IL-2 and IL-12) and type 2 (IL-4, IL-5 and IL-10) cytokines. As shown in Figure 1C, type 1 and type 2 cytokines were present in the lungs of both studied groups, but at the early phase of infection only TNF-α appeared in significantly higher levels in NO-deficient mice. Later in the infection, however, no differences in pulmonary cytokines were detected (Figure 1D). These data suggested that TNF-α could be involved in the early immunoprotection conferred by NO deficiency.

### iNOS deficiency induces increased migration of activated macrophages and lymphocytes into the lungs

**Figure 2:**
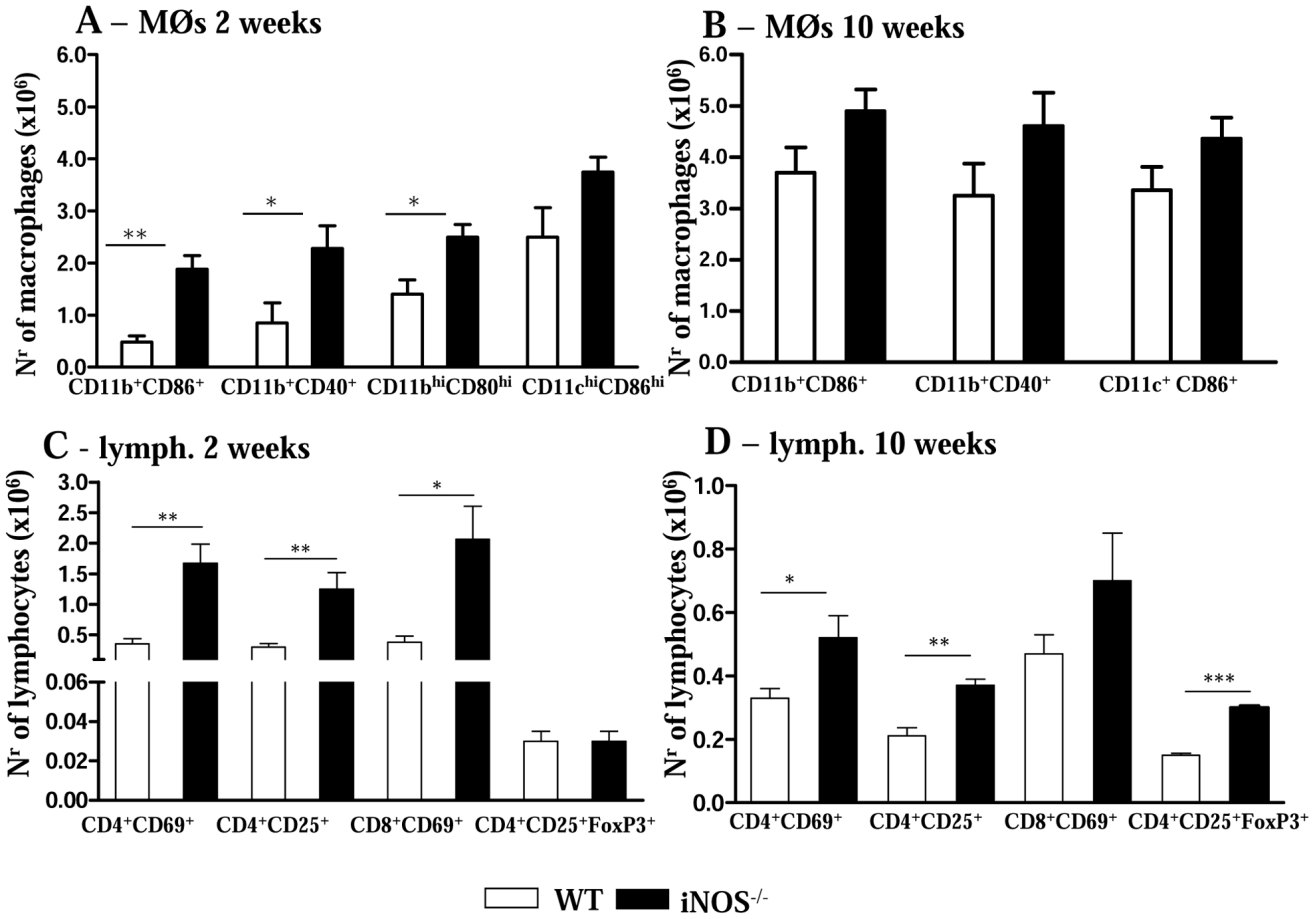
iNOS activity controls the influx of activated mononuclear phagocytes and T cells to the lungs. Flow cytometry characterization of lung infiltrating leucocytes (LIL) from iNOS^−/−^ and WT mice after i.t. infection with 1×10^6^ *P. brasiliensis* yeast cells. Lungs of iNOS^−/−^ and WT mice (n = 6–8) were excised, washed in PBS, minced, and digested enzymatically. At weeks 2 (A, C) and 10 (B, D) after infection, lung cell suspensions were obtained and stained as described in Materials and Methods. (A, B) Phenotypic characterization of CD11b^+^ mononuclear phagocytes expressing CD80, CD86 and CD40 and dendritic cells expressing high levels of CD11c and CD86 (CD11c^high^CD86^high^). An increased presence of mononuclear phagocytes was observed in deficient mice, while the number of dendritic cells was equivalent in the lungs of both mouse strains. (C, D) Characterization of T cell subsets by flow cytometry in LIL obtained at weeks 2 (C) and 10 (D) after infection. To characterize the expansion of regulatory T cells in LIL, surface staining of CD25^+^ and intracellular FoxP3 expression were back-gated on the CD4^+^ T cell population. The acquisition and analysis gates were restricted to macrophages (A, B) or lymphocytes (C, D). The data represent the mean ± SEM of the results from 6–8 mice per group and are representative of two independent experiments. * (*P*<0.05), ** (*P*<0.01) and *** (*P*<0.001), compared with WT mice.

We have also evaluated the presence and the activation profile of leukocytes in the lungs from both mouse strains. A higher number of mononuclear phagocytes expressing activation molecules was detected in iNOS^−/−^ mice when compared with WT mice. As shown in Figure 2A, at the 2^nd^ week, the number of double positive CD11b^+^CD86^+^ and CD11b^+^CD40^+^ cells was higher in iNOS^−/−^ mice. Further phenotypic characterization of macrophages (CD11b^high^CD80^high^) and dendritic cells (CD11c^high^CD86^high^) demonstrated the increased presence of macrophages in the deficient mouse strain, while the number of dendritic cells was equivalent in both studied groups (Figure 2A). By week 10, no significant differences in the number of pulmonary macrophages were detected (Figure 2B).

In addition, to determine the lymphocyte influx and the activation profile of CD4^+^ and CD8^+^ T cells in the lungs of *P. brasiliensis* infected mice, we evaluated the expression of CD69 and CD25 by freshly isolated T cells. The marker CD69 is a very early activation antigen [41] as well as CD25, the α-chain of the interleukin-2 receptor [42], which is rapidly upregulated on activated T cells. Compared with the control group, at the 2^nd^ week, NO-deficient mice presented an increased recruitment of activated CD4^+^CD69^+^, CD4^+^CD25^+^ and CD8^+^CD69^+^ T lymphocytes to the lungs (Figure 2C). Although in lower intensity, at the chronic phase, the recruitment/expansion of CD4^+^CD69^+^ and CD4^+^CD25^+^ T cells to the lungs of iNOS^−/−^ mice remained higher than in WT mice. The same was not verified for CD8^+^CD69^+^ T cells that appeared in equivalent numbers in both mice strains (Figure 2D). Because Treg cells control the expansion of effector T cells, and the number and function of these cells were shown to be influenced by NO production [43], [44] we characterized the presence of CD4^+^CD25^+^FoxP3^+^ T cells in the pulmonary cell infiltrates (Figure 2C and 2D). Although at the week 2, no differences in the numbers of Treg cells were seen, by week 10 increased numbers of CD4^+^CD25^+^FoxP3^+^ cells were detected in iNOS^−/−^ mice. This finding paralleled the increased pulmonary CFU counts, the diminished number of T cells and the impaired macrophage activation detected in the lungs of iNOS^−/−^ mice at this late period of the infection.

### Studies on the killing ability of alveolar macrophages from iNOS^−/−^ and WT mice

**Figure 3:**
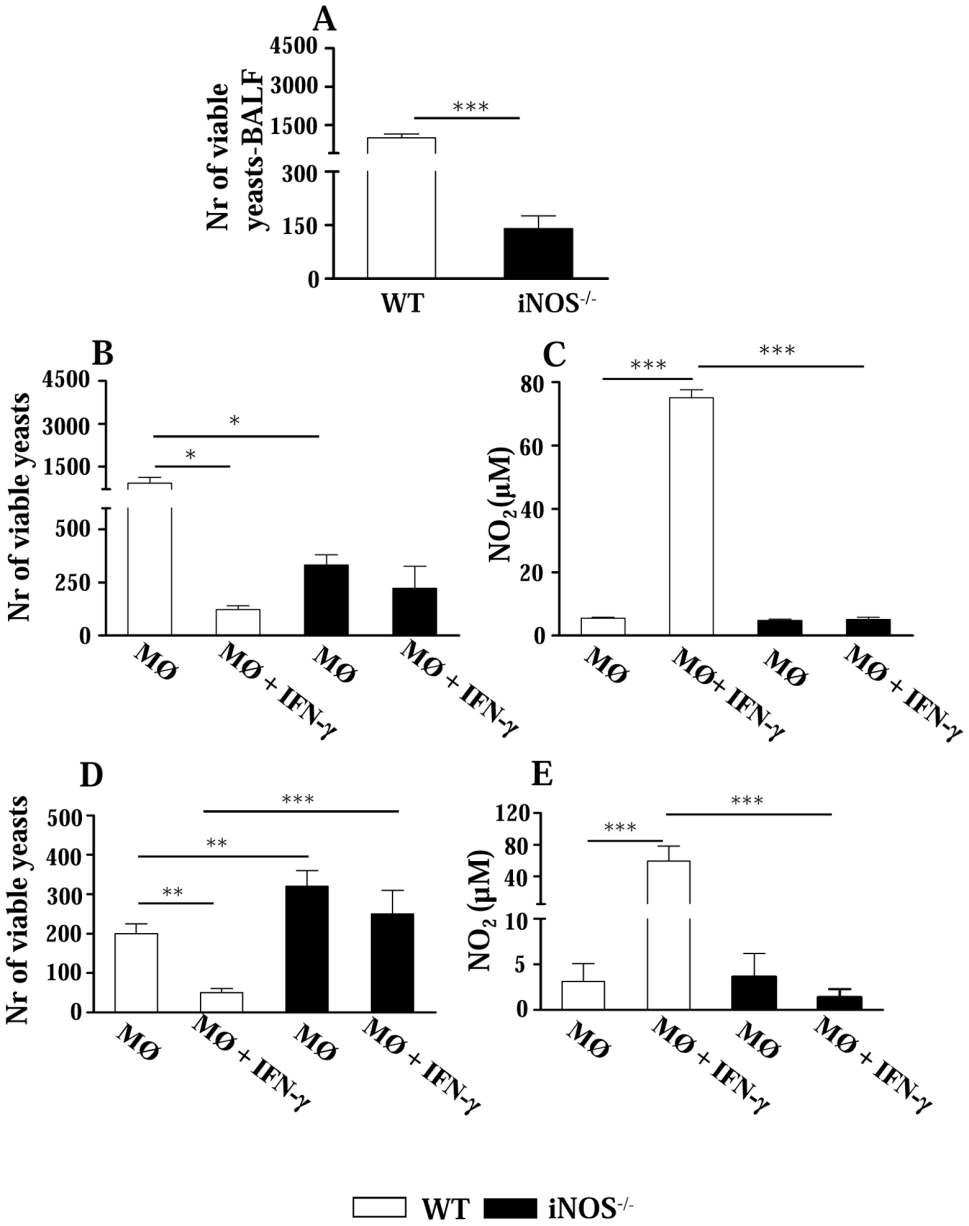
Fungal loads and fungicidal activity of alveolar macrophages. Early in infection (week 2), bronchoalveolar lavage fluids (BALFs) of iNOS^−/−^ mice have a lower fungal load, but alveolar macrophages are refractory to IFN-γ activation. (A) Number of viable yeasts (CFU counts) recovered from BALFs of iNOS^−/−^ and wild type (WT) C57BL/6 mice, 2 weeks after *P. brasiliensis* infection with 1×10^6^ yeast cells. (B and D) Viable yeasts recovered from adherent alveolar macrophages obtained at weeks 2 and 10 after infection, respectively, stimulated or not by IFN-γ (100 U/ml) and cultivated *in vitro* for 48 h. (C and E) Levels of NO in the supernatants of alveolar macrophages. The bars depict means ± SEM (6–8 animals per group) and are representative of three experiments. * (*P*<0.05), ** (*P*<0.01), and *** (*P*<0.001) compared with WT controls.

After characterizing the main features of the infection, we aimed to clarify the mechanisms involved in the early immunoprotection conferred by iNOS deficiency. Thus, the behavior of alveolar macrophages was assessed at two opposed periods of infection. In agreement with lung CFU data, at the 2^nd^ week a lower number of yeasts was recovered from the bronchoalveolar lavage fluid of iNOS^−/−^ mice (Figure 3A). The microbicidal activity of alveolar macrophages was further determined after 48 h cultivation in the presence or absence of IFN-γ. Again, lower CFU counts were recovered from deficient macrophages (Figure 3B). As expected, only WT macrophages showed decreased fungal counts associated with increased NO production after IFN-γ treatment (Figure 3B and 3C, respectively). At week 10, iNOS-deficient macrophages showed higher fungal loads than WT cells, which increased their fungicidal ability after IFN-γ treatment (Figure 3D and 3E).

### INOS^−/−^ macrophages are refractory to IFN-γ and TNF-α activation, have an impaired phagocytic activity, and express high levels of TGF-β and arginase 1

**Figure 4:**
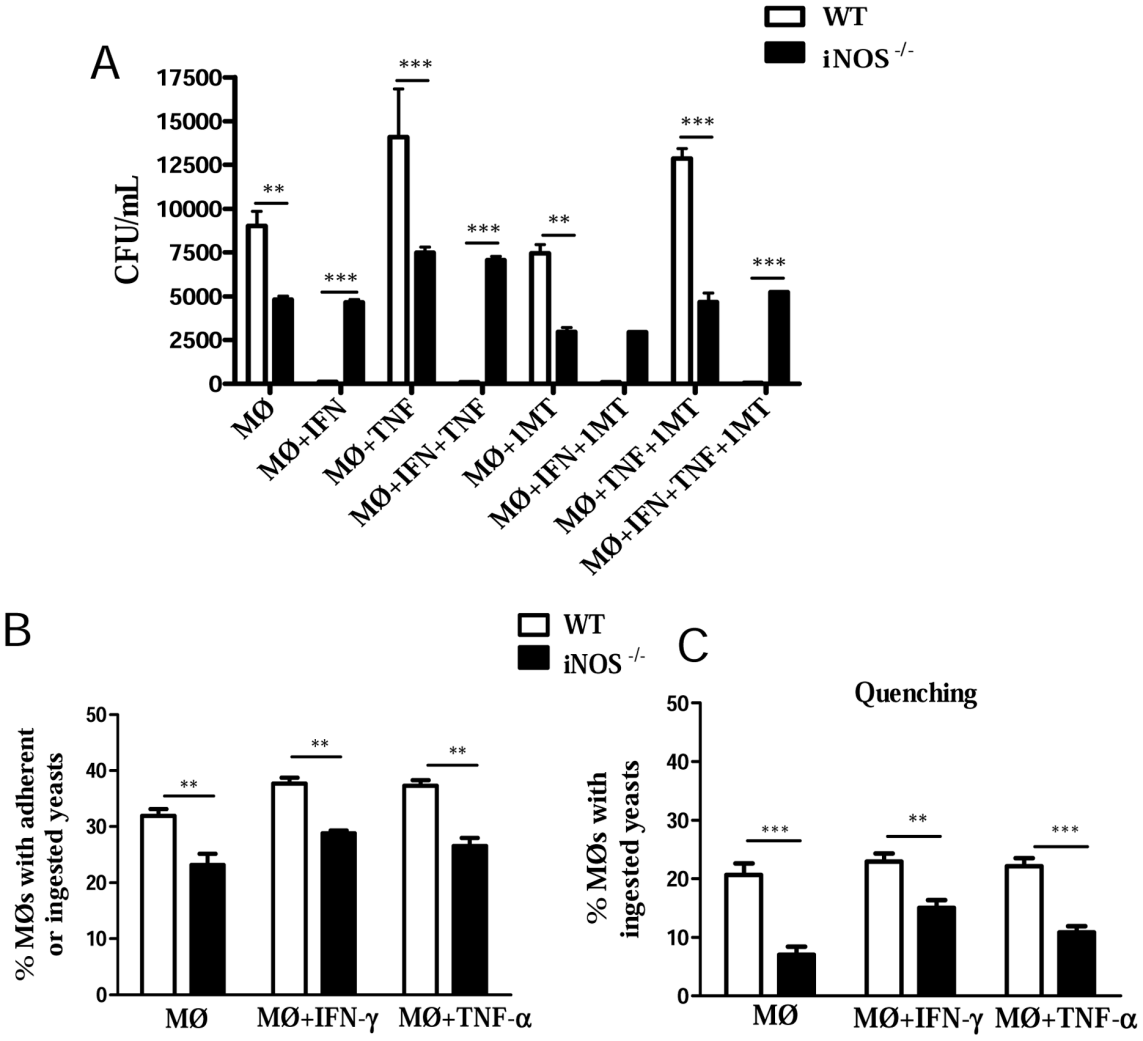
INOS^−/−^ macrophages are refractory to IFN-γ and TNF-α activation and have an impaired phagocytic activity. (A) For fungicidal assays, peritoneal macrophages from WT and iNOS ^−/−^ C57BL/6 mice were cultivated in the presence or absence of recombinant IFN-γ (40 ng/ml), TNF-α (20 ng/ml) or 1-methyl-DL-tryptophan (1 MT, 1 mM), a specific inhibitor of 2,3 indoleamine dioxygenase. Cultures were infected with *P. brasiliensis* yeasts in a macrophage∶yeast ratio of 12.5∶1. After 48 h, macrophages were lysed, and viable yeasts determined using a CFU assay. All analyzes were done with five wells per condition in three independent experiments. (B) For phagocytic assays, macrophages were infected with heat-inactivated, FITC labeled, *P. brasiliensis* yeasts at a macrophage∶yeast ratio of 1∶1 for 2 h at 37°C in 5% CO_2_ to allow fungi adhesion and ingestion. Some macrophage cultures were treated with IFN-γ (40 ng/ml), or TNF-α (20 ng/ml) overnight, before infection. Macrophages were washed, cells detached from plastic, and labeled with anti-F4/80 (APC) antibodies. The cell suspensions were immediately read on a FACScalibur cytometer. All analyzes were done with five wells per condition in three independent experiments. (C) A quenching assay employing a trypan blue solution (TB, 250 µg/mL) was used to distinguish internalized from surface-bound yeasts (FITC- labeled *P.brasiliensis* particles). Phagocytic assays were performed as above described, and adherent/ingested cells measured using the FL1 and FL4 channels of a FACscalibur cytometer. Cell suspensions were then treated with a TB solution for quenching the green surface-bound fluorescence on macrophages and samples were again analyzed. APC-labeled macrophages were gated, and FL1 and FL3 channels used to discriminate ingested (green fluorescent, FL1) from adherent (red fluorescent, FL3) yeasts. All analyzes were done with five wells per condition in three independent experiments. The bars depict means ± SEM ** (*P*<0.01) and *** (*P*<0.001) compared with WT controls.

Studies with alveolar macrophages raised two important questions. Were the decreased fungal loads of iNOS^−/−^ macrophages due to their increased fungicidal activity or decreased phagocytic ability? Why are iNOS^−/−^ macrophages refractory to IFN-γ activation? Therefore, inflammatory peritoneal macrophages were used to better understand the behavior of iNOS-deficient phagocytes. Peritoneal macrophages were obtained, activated or not by IFN-γ, TNF-α or both cytokines and infected by *P. brasiliensis*. Some activated and non-activated cells were also treated with 1MT, a specific inhibitor o 2,3 indoleamine dioxygenase, an enzyme that catalyzes the degradation of tryptophan along the kynurenine pathway. In fungal infections, this enzyme was shown to exert an efficient fungicidal activity but also an important suppressive effect on the immune response [45]. Recapitulating the results obtained with alveolar macrophages, lower numbers of viable yeasts were recovered from iNOS^−/−^ macrophages (Fig. 4A). Only WT cells increased their fungicidal ability when activated by IFN-γ, although TNF-α was not able to modify the fungicidal activity of cells from both mouse strains. Similar result was observed with 1MT-treated macrophages. The IDO inhibitor was not able to modify the behavior of activated and non-activated macrophages from WT and iNOS^−/−^ mice (Fig. 4A).

We have then used FITC-labeled *P. brasiliensis* yeasts to discriminate adherent and ingested cells. As shown in Fig. 4B, iNOS^−/−^ macrophages have reduced capacity to adhere/ingest fungal cell. When the green fluorescence of adhered yeasts was quenched by trypan blue treatment, a decreased ingestion activity was observed with WT and iNOS-deficient macrophages, although more evident with the latter cells. Thus, iNOS^−/−^ macrophages have a reduced ability to adhere and ingest fungal cells, and this behavior appears to explain the reduced CFU counts detected in vitro and in vivo with iNOS^−/−^ cells.

**Figure 5:**
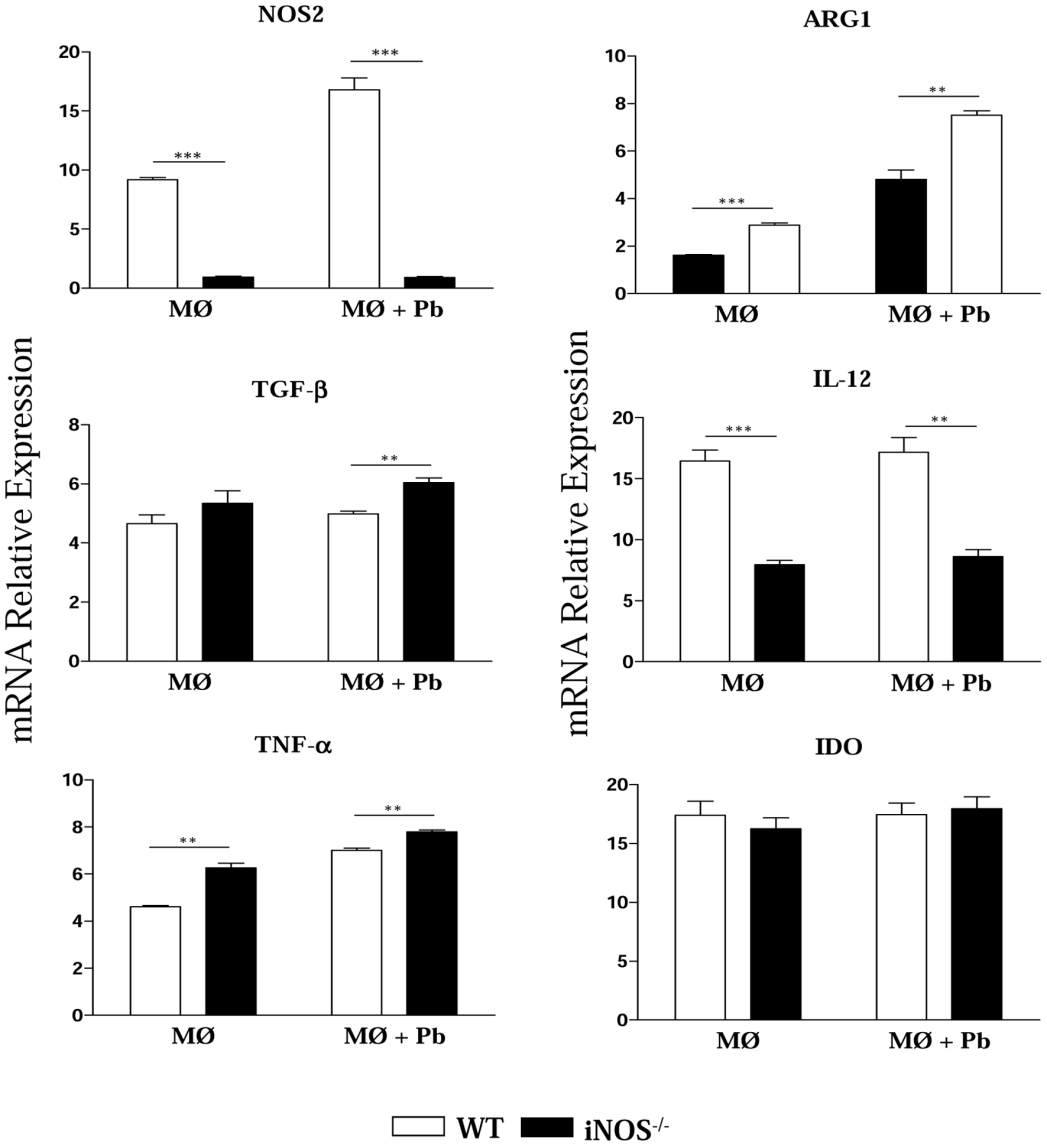
INOS^−/−^ macrophages express high levels of TGF-β arginase1 and TNF-α. Uninfected and *P. brasiliensis* infected macrophages were used to characterize the expression of iNOS, ARG1, TGF-β, IL-12, TNF-α and IDO mRNA by quantitative Real-Time PCR. Total RNA was extracted using Trizol reagent, reverse transcribed, and cDNA amplified. Real-time PCR was performed using TaqMan universal master mix. Amplified products were normalized to the amount of GAPDH products from macrophages. All analyzes were done with five wells per condition in three independent experiments. The bars depict means ± SEM ** (*P*<0.01) and *** (*P*<0.001) compared with WT controls.

To better characterize the differentiation of iNOS^−/−^ and WT macrophages, the expression of iNOS, ARG1, TGF-β, IL-12, TNF-α and IDO mRNA was measured in uninfected and *P. brasiliensis* infected cells (Fig. 5). iNOS-deficient macrophages showed reduced expression of IL-12 associated with increased production of ARG1 and TGF-β mRNA, demonstrating a prevalent anti-inflammatory behavior and some characteristics of M2, healing, or alternatively activated macrophages. An opposite result was observed with WT macrophages which expressed high levels of iNOS and IL-12 mRNA. Interestingly, no differences in IDO expression were detected between WT and iNOS^−/−^ cells, but the latter showed increased expression of TNF-α.

### iNOS-deficient mice develop increased DTH reactions, more organized lung lesions and decreased mortality rates

**Figure 6:**
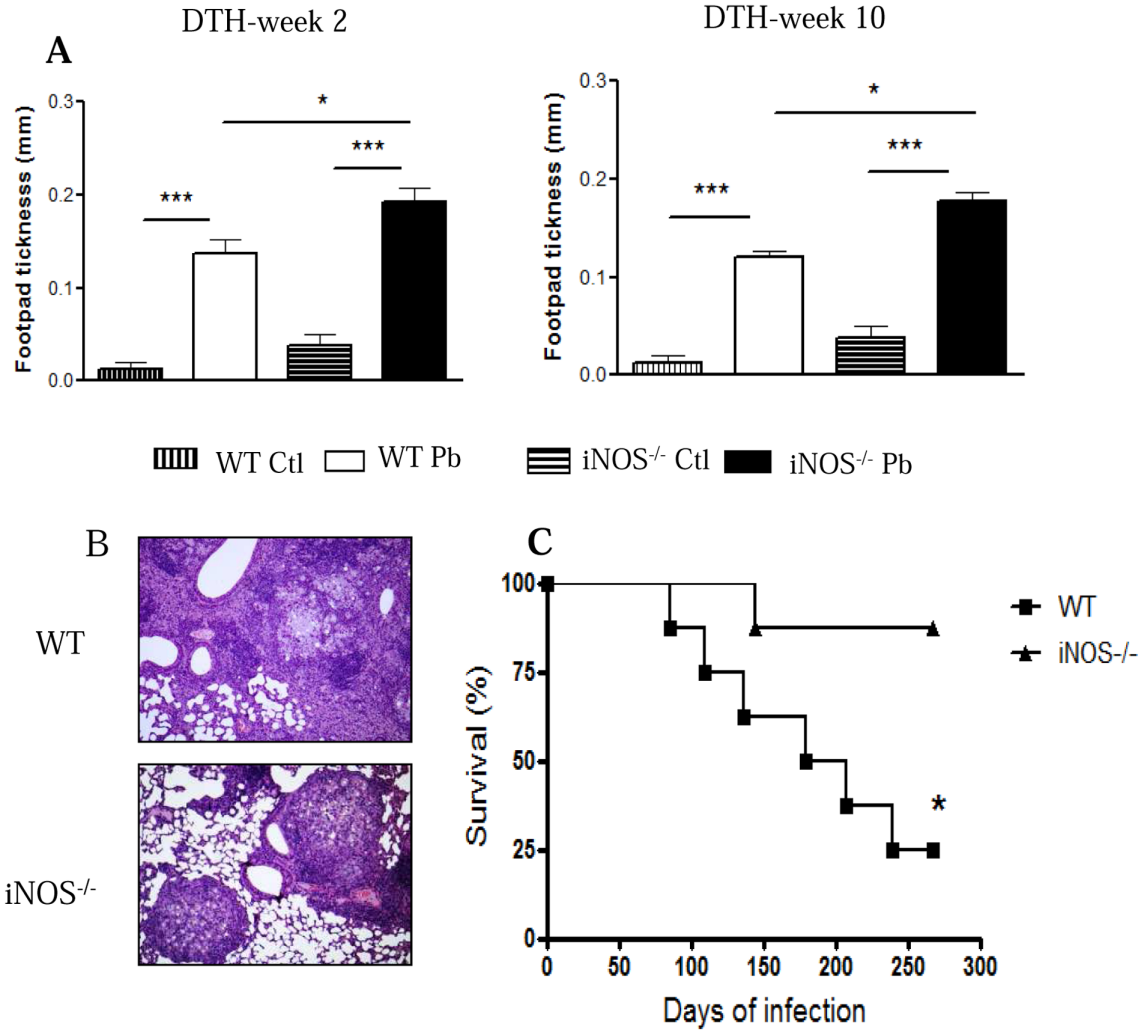
iNOS−/− mice show a better disease outcome than WT mice. Positive DTH reactions and well-organized lesions appear to compensate the increased fungal burdens, and are associated with increased survival times of iNOS^−/−^ mice. (A) iNOS ^−/−^ and WT mice were i.t. infected with 1×10^6^ *P.brasiliensis* yeasts and DTH reactions measured 2 and 10 weeks later. Uninfected mice were used as control. DTH reactions were evaluated in control and infected mice by the footpad swelling 48 h after the s.c. injection of 25 µl of a soluble *P. brasiliensis* antigen. The bars depict means ± SE of footpad swelling. (n = 6–8). (B) Photomicrographs of pulmonary lesions of WT (Fig. 4B, upper panel), and iNOS^−/−^ (Fig. 4B, lower panel) at weeks 10 of infection with 1×10^6^ *P. brasiliensis* yeasts. Both mouse strains developed extensive lesions, but organized lesions were only observed in NO-deficient mice (HE, 100×). (C) Survival times of iNOS^−/−^ and WT control mice (n = 6–8) after i.t. infection with 1×10^6^ *P. brasiliensis* yeast cells was determined in a period of 300 days. The data are representative of three independent experiments with equivalent results. * (*P*<0.05), and *** (*P*<0.001) compared with WT controls.

The delayed type hypersensitivity (DTH) reactions developed by iNOS^−/−^ and WT mice were evaluated at weeks 2 and 10 after infection using a soluble *P.brasiliensis* antigen. As depicted in Figure 6A, both mouse strains developed increased footpad reactions, but these were significantly higher in iNOS^−/−^ than in WT mice. To better typify the effect of iNOS deficiency in the severity of pulmonary PCM, histopathological analysis of lung sections from iNOS^−/−^ and WT mice at both periods of infection was also performed. By week 2, iNOS^−/−^ and WT mice showed diffuse inflammatory reactions composed of monocytes and lymphocytes surrounding interlobular spaces localized around the bronchi, bronchioles and blood vessels. However, an increased presence of inflammatory cells accompanied the lower CFU counts observed in the lungs of iNOS^−/−^ mice (data not shown). Surprisingly, at week 10 remarkable histopathological differences were detected; iNOS^−/−^ mice presented a large number of well-organized granulomas (Figure 6B, lower panel) containing an elevated number of yeast cells surrounded by epithelioid and multinuclear giant cells, and a well-defined lymphocytic mantle. Plasma cells and eosinophils were scarce. The fungi, detected in great numbers, were large and have multiple buds. Compared with iNOS^−/−^ mice, WT mice presented more extensive, non-organized lesions, and decreased fungal loads irregularly distributed in the lung parenchyma (Figure 6B, upper panel). Importantly, despite the higher number of yeasts recovered late in the infection, iNOS^−/−^ mice showed decreased mortality rates (Figure 6C). Thus, the increased T cell immunity as evidenced by increased DTH reactions, and the more organized lesions (possibly mediated by the increased TNF-α secretion) appear to have compensated the poor control of fungal multiplication resulting from iNOS deficiency

### In vivo depletion of TNF-α does not increase fungal loads but induces increased recruitment of inflammatory cells to the lungs

**Figure 7:**
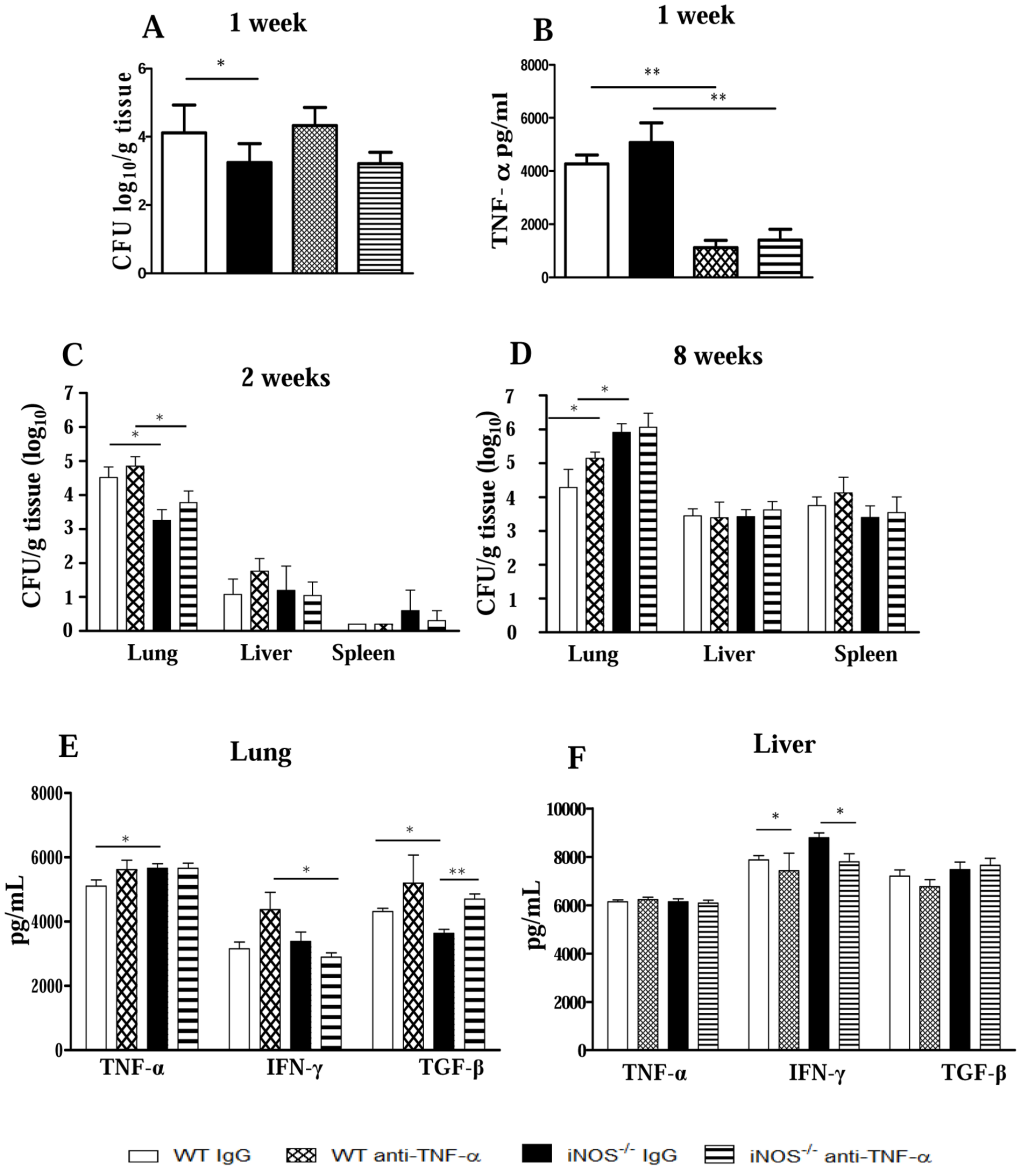
Neutralization of TNF-α does not alter fungal loads and has a minor effect on organ cytokines. WT and iNOS^−/−^ mice (n = 6–7) were treated with anti-TNF-α mAb (MP6 XT 22, i.p. injections of 0.25 mg/0.5 mL) 4 h before, and at days 6 and 12 of infection). Normal rat IgG was used as control. CFU counts were determined at weeks 1, 2 and 8 postinfection with 1×10^6^ *P.brasiliensis* yeasts. (A, C, and D, respectively). Levels of pulmonary (B, E) and hepatic (F) cytokines were assessed in organ homogenates obtained at the 1^st^ (B) or 2^nd^ (E, F) weeks postinfection. The data represent the mean ± SEM of the results from 6–7 mice per group and are representative of two independent experiments. * (*P*<0.05) and ** (*P*<0.01).

Supported by the remarkable increase of TNF-α levels observed early in the infection in the lungs of iNOS^−/−^ mice, and to further understand the mechanisms of immunoprotection used by this mouse strain to control *P.brasiliensis* infection, iNOS^−/−^ and WT groups were *in vivo* depleted of TNF-α and the severity of infection analyzed by CFU counts and pulmonary inflammation. At week 1 after infection, a significant difference in fungal burdens were observed between IgG-treated WT and iNOS^−/−^ mice. The anti-TNF-α treatment, however, abolished this difference (Figure 7A). Anti-TNF-α treatment was shown to be effective, since reduced levels of this cytokine were detected in the treated groups of both mouse strains (Figure 7B). In addition, in both mouse strains at weeks 2 and 8 after infection TNF-α depletion did not result in significant increases in pulmonary fungal burdens (Figure 7C, D). Yet, no significant differences were noticed in the dissemination to liver and spleen. Cytokines measurements in lung homogenates at week 2 after infection demonstrated that, although anti-TNF-α treatment remained until day 12 postinfection, TNF-α rapidly reached the pre-treatment levels in the liver and lungs of both mouse strains (Figure 7E and 7F). On the other hand, a higher concentration of TGF-β was detected in the lungs, while decreased levels of hepatic IFN-γ were seen in anti-TNF-α treated iNOS^−/−^ mice (Figure 7E, F).

### Anti-TNF-α treatment results in exacerbated lung inflammation and unorganized pulmonary lesions in both mouse strains, but increased mortality only in iNOS^−/−^ mice

**Figure 8:**
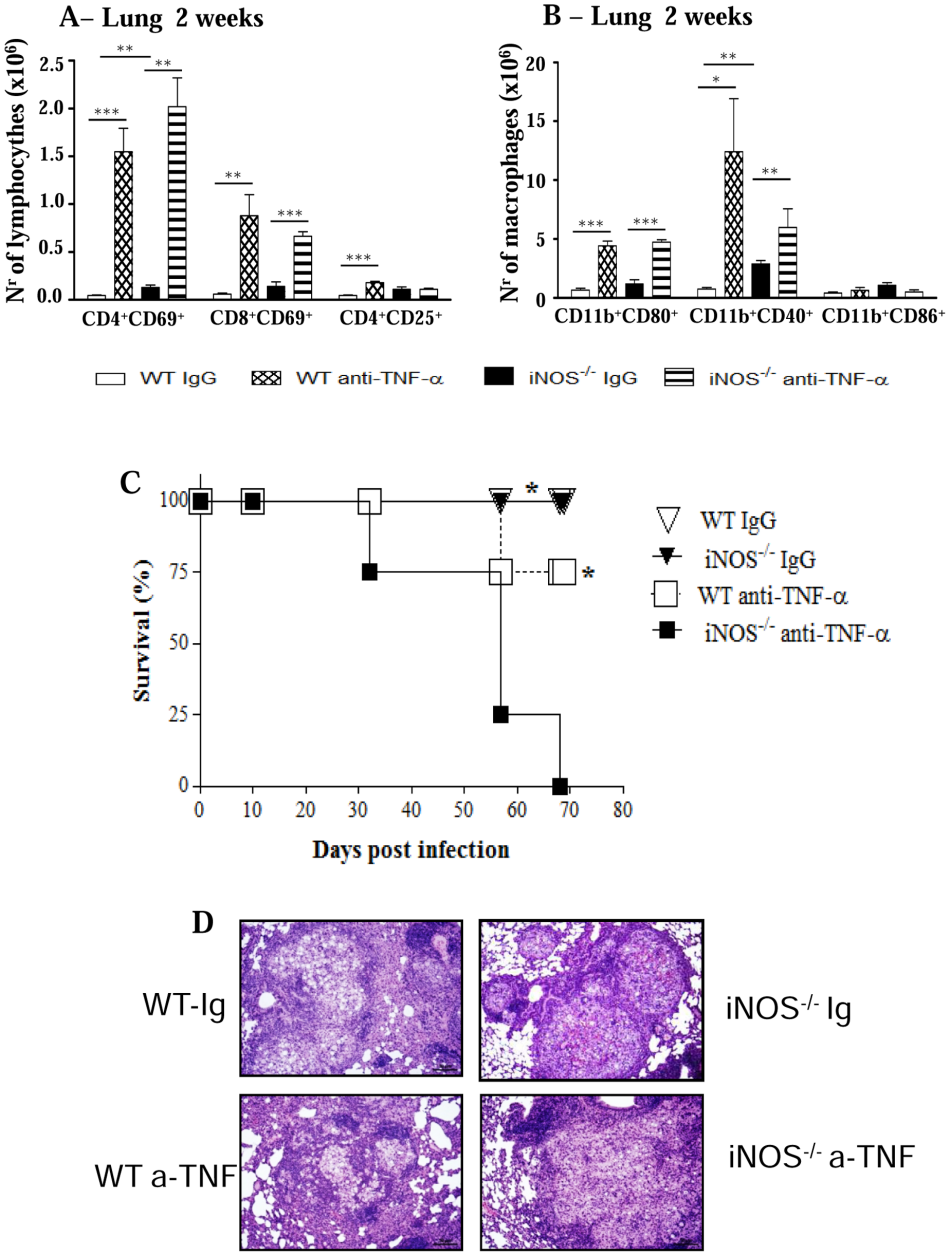
Neutralization of TNF-α is more detrimental to iNOS^−/−^ than WT mice. TNF-α neutralization leads to increased influx of activated cells to the lungs, increased mortality and non-organized lesions in iNOS-deficient mice. WT and iNOS^−/−^ mice were treated with anti-TNF-α mAb (MP6 XT 22) or normal rat IgG (control) and i.t. infected with 1×10^6^ fungal cells. Number and activation of lymphocytes (A) and mononuclear phagocytes (B) present in pulmonary lesions at the 2^nd^ week postinfection of anti-TNF-α treated and untreated mice were determined by flow cytometry. (C) Survival times of anti-TNF-α treated and untreated mice. TNF-α depleted iNOS^−/−^ mice showed a decreased survival time, which was significantly different (* *P*<0.05) from all other studied groups. The results are representative of three independent experiments (n = 6–7). (D) Photomicrographs of pulmonary lesions developed by IgG-treated (D, superior panels) and TNF-α-depleted (B, lower panels) mice at the week 8 of infection. Anti-TNF-α treatment of iNOS-deficient mice resulted in less organized, fungi rich, confluent lesions, occupying the largest part of lung tissue. H&E stained lesions (100×).

Unexpectedly, when lung infiltrating leukocytes were characterized at week 2 postinfection, an impressive increase in the numbers of lymphocytes (Figure 8A) and mononuclear phagocytes (Figure 8B) were seen in the lungs of anti-TNF-α-treated mice. Thus, a higher influx of activated CD4^+^CD69^+^, CD8^+^CD69^+^ T cells into the lungs of TNF-depleted WT and iNOS^−/−^ mice was observed, although CD4^+^CD25^+^ T appeared in higher numbers only in the former strain (Figure 8A). In addition, compared with IgG-treated controls, iNOS^−/−^ and WT-depleted groups showed elevated numbers of CD11b^+^CD80^+^ and CD11b^+^CD40^+^ mononuclear phagocytes (Figure 8B).

To further characterize the role of TNF-α in our model, TNF-α-depleted and IgG-treated controls were studied regarding mortality and histopathology of lungs. As depicted in Figure 8C, the effect of TNF-α neutralization was much more prominent in the iNOS-deficient strain. Indeed, 100% of TNF-α depleted iNOS^−/−^ mice died within 70 days of infection while only 25% of WT mice died in the same period. This result demonstrated that, at least partially, the relative protection of NO-deficient mice was due to the enhanced TNF-α production. In vivo neutralization of TNF-α did not appear to cause clear alterations in the already poor-organized and confluent lesions of WT mice (Figure 8D, left panels). As described for untreated-infected mice, IgG-treated iNOS^−/−^ mice presented better-defined granulomas, and a high influx of inflammatory cells, which appears to restrain fungal spreading (Figure 8D, upper right panel). In contrast, anti-TNF-treated iNOS^−/−^ mice lose this organized pattern of lesions and non-organized, confluent inflammatory reactions containing fungal cells were scattered through the pulmonary tissue (Figure 8D, lower right panel).

### Depletion of CD8α ^+^ cells abolishes the differences in the early fungal loads and inflammatory reactions conferred by iNOS deficiency

**Figure 9:**
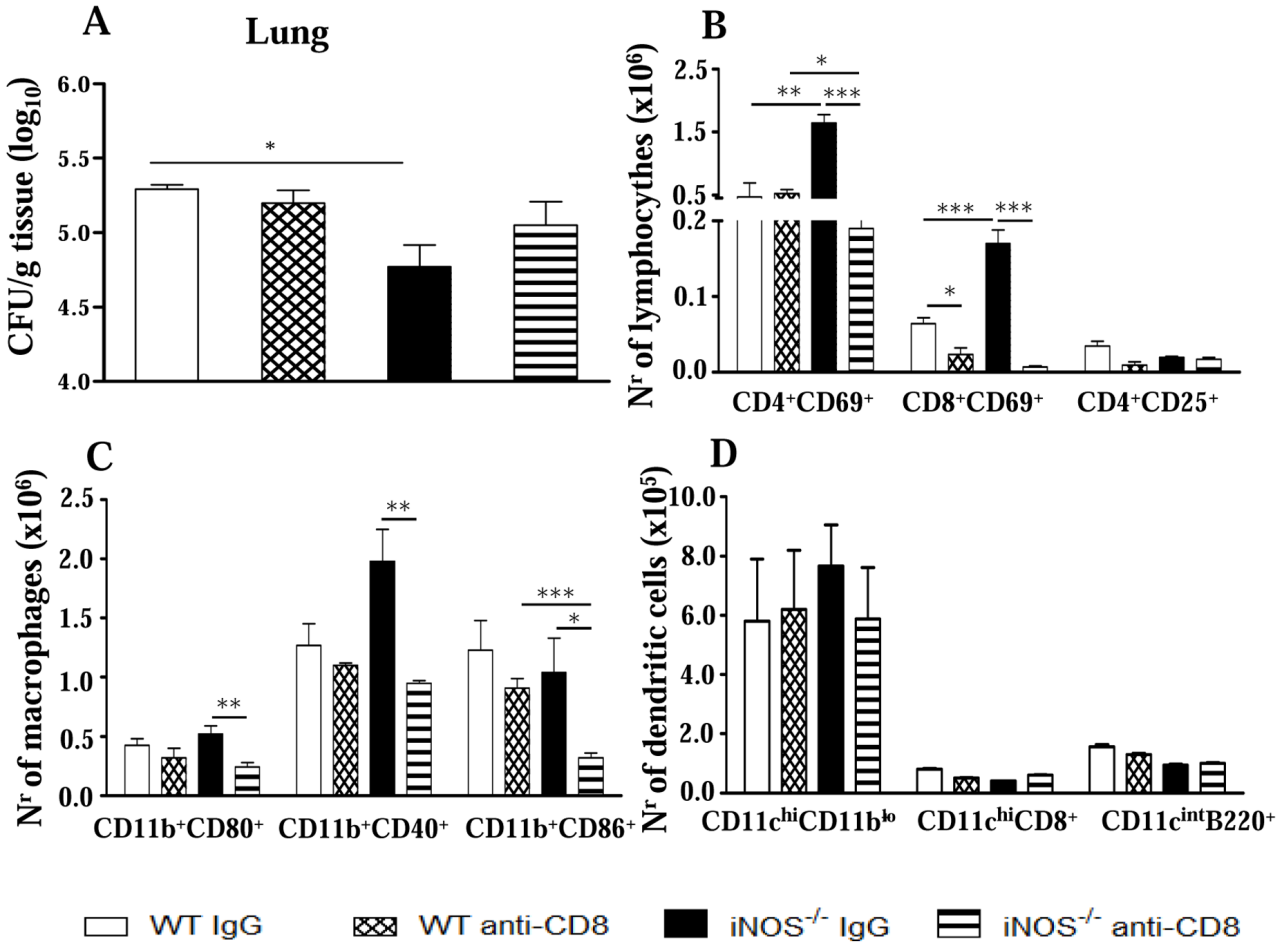
CD8^+^ T cells control the early influx of inflammatory cells to the lungs of iNOS-deficient mice. Groups (n = 6–7) of WT and iNOS^−/−^ mice were given 200 µg of anti-CD8α mAbs (H-35) or normal rat IgG (controls) by the i.p. route, 48 and 24 h before infection, and 150 µg of the anti-CD8α mAbs or rat IgG at days 6 and 12 of infection with 1×10^6^ *P. brasiliensis*. The severity of infection was assessed by organ CFU counts (A) at the 2^nd^ week of infection. The phenotypes of lung infiltrating lymphocytes (B), mononuclear phagocytes (C), and CD11c^+^ dendritic cells isolated by magnetic beads (D) were characterized by flow cytometry. The results are representative of two experiments. * (*P*<0.05), ** (*P*<0.01), and *** (*P*<0.001).

The lower pulmonary fungal growth detected at week 2 postinfection of iNOS^−/−^ mice paralleled the enhanced presence of inflammatory cells (Figures 1A and 2C). Importantly, among T cells, only the CD8^+^ T cell subset lost their activation profile at week 10 of infection when iNOS^−/−^ mice were unable to control of fungal growth. In order to study the involvement of CD8^+^ T cells in the early immunoprotection of NO-deficient mice, CD8α^+^ cells were in vivo depleted by monoclonal antibodies, and the severity of infection characterized at week 2 postinfection. As depicted in Figure 9A, anti-CD8 treatment abolished the differences in CFU counts previously observed in the lungs of mice. Moreover, this treatment also abolished the higher presence of activated T cells and macrophages observed in the lungs of iNOS-deficient mice at this early period of infection. Compared with IgG-treated controls, CD8-depleted iNOS^−/−^ mice presented significantly diminished influx of CD4^+^CD69^+^ and CD8^+^CD69^+^ T cells to the lungs (Figure 9B). The same occurred with CD11b^+^CD80^+^, CD11b^+^CD40^+^ and CD11b^+^CD86^+^ mononuclear phagocytes (Figure 9C).

Subsequently, we asked whether this difference in cell influx caused by anti-CD8α treatment was due to the depletion of the CD8α^+^ subset of dendritic cells. Thus, IgG-treated and anti-CD8-depleted WT and iNOS^−/−^ groups were i.t. infected and at the second week post-infection the lungs were removed, digested and pulmonary dendritic cells obtained with anti-CD11c magnetic beads. The phenotype of these cells was then assessed by flow cytometry. The Figure 9D demonstrates that no differences in the numbers of conventional (CD11c^high^CD11b^low^), lymphoid (CD11c^+^CD8α^+^) and plasmacytoid (CD11c^+^B220^+^) DCs were detected between anti-CD8-treated and untreated mice of both mouse strains. In summary, this result suggested that the effect of anti-CD8 treatment was mainly mediated by CD8α^+^ T lymphocytes and not lymphoid DCs.

### Anti-CD8 treatment diminishes the number of TNF-α and IFN-γ secreting cells in the lungs of NO-deficient mice

**Figure 10:**
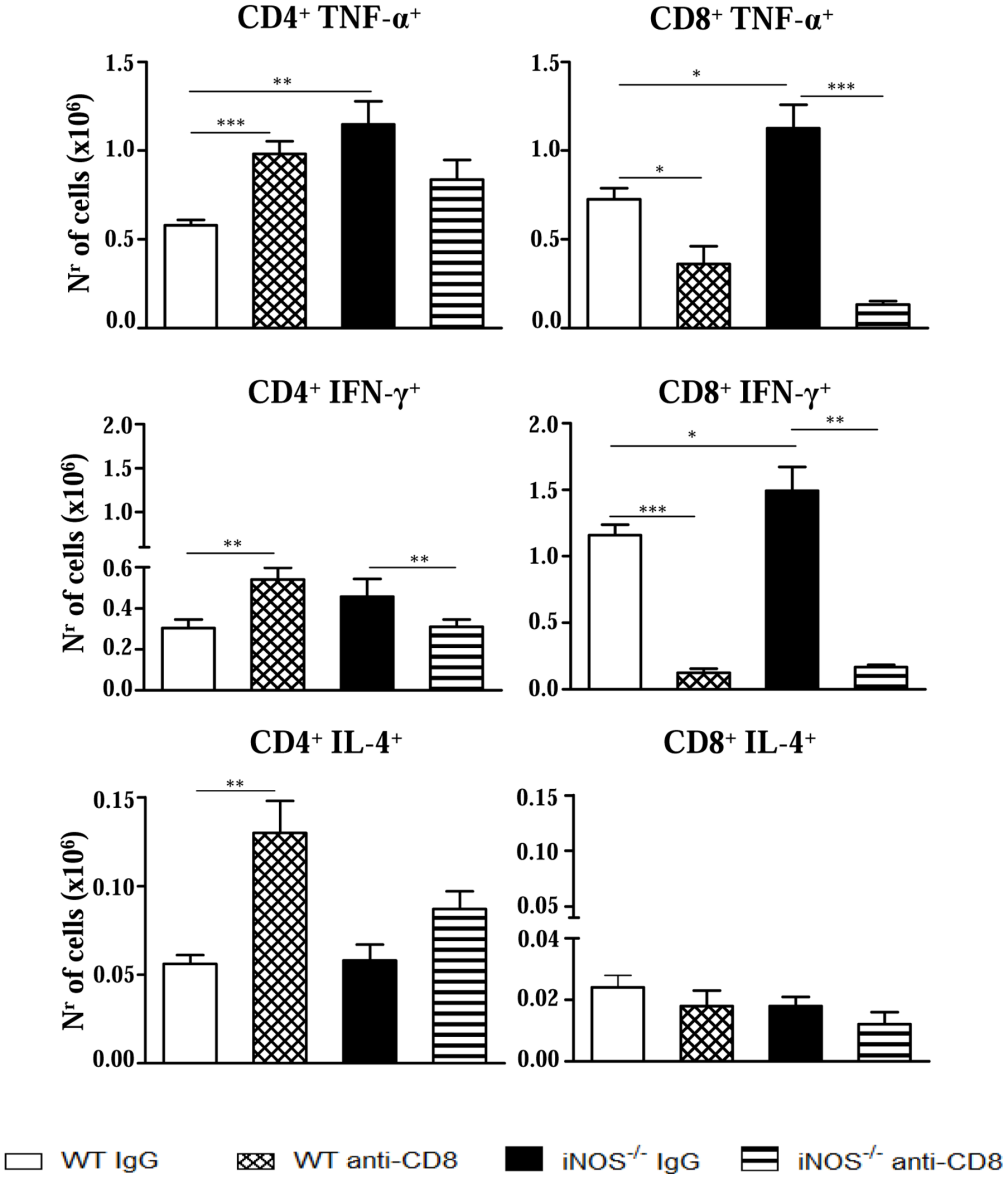
Depletion of CD8^+^ T cells alters the numbers of TNF-α^+^, IFN-γ^+^ and IL-4^+^ T cells in the lungs of iNOS^−/−^ and WT mice. Groups (n = 6–7) of WT and iNOS^−/−^ mice were treated with 200 µg of anti-CD8α mAbs (H-35) or normal rat IgG (controls) and i.t. infected with 1×10^6^ *P. brasiliensis*. The presence of TNF-α^+^, IFN-γ^+^ and IL-4^+^ CD4^+^ and CD8^+^ T cells in the lung infiltrating leukocytes was assessed by intracellular cytokine staining by flow cytometry at week 2 after infection. Lung cells were re-stimulated in vitro with PMA/ionomycin for 6 h and subjected to intracellular staining for TNF-α, IFN-γ and IL-4. The lymphocyte population was gated by forward/side scatters. Results are from one experiment and are representative of two independent experiments. * (*P*<0.05), ** (*P*<0.01), and *** (*P*<0.001).

Next, we characterized the phenotype of IFN-γ, TNF-α and IL-4-secreting lymphocytes in the lungs of anti-CD8-treated and IgG-treated iNOS^−/−^ and WT mice by intracellular cytokine staining. Comparing IgG-treated controls, enhanced numbers of TNF-α ^+^ CD4^+^ and CD8^+^ T cells were seen in the lungs of iNOS^−/−^ than in the WT group (Figure 10). Anti-CD8 treatment led to increased numbers of TNF-α^+^, IFN-γ^+^ and IL-4^+^ CD4^+^ T cells in WT mice. However, reduced numbers of TNF-α^+^ and IFN-γ^+^ CD8^+^ T cells appeared in the lungs of this strain. Importantly, in iNOS^−/−^ mice the depletion of CD8^+^ cells resulted in a remarkable reduction of TNF-α^+^ and IFN-γ^+^ CD8^+^ T cells, besides a decreased presence of IFN-γ^+^ CD4^+^ T cells (Figure 10). Therefore, at the 2^nd^ week of *P. brasiliensis* infection, the reduction of pro-inflammatory CD8^+^ T cells observed in WT mice appeared to be compensated by the increased presence of TNF-α- and IFN-γ-secreting CD4^+^ T cells whereas in iNOS-deficient mice a prevalent reduction of pro-inflammatory CD4^+^ and CD8^+^ T cells was seen.

## Discussion

In the present work, we investigated the temporal significance of nitric oxide synthesis in the evolution of pulmonary paracoccidioidomycosis and the immunopathological mechanisms associated with iNOS deficiency. Early in infection, the protective effects of iNOS deficiency was associated with decreased fungal burdens, enhanced secretion of TNF-α augmented DTH reactions, and increased migration of activated T cells and macrophages to the lungs, which subsequently organize as well-compact granulomas. On the other hand, at later periods, increased fungal loads were concomitant with sustained T cell immunity allied with increased presence of regulatory T cells at the site of infection. Unexpectedly, the mortality rates of WT mice were higher than those of iNOS^−/−^ mice. In vivo depletion of TNF-α and CD8^+^ T lymphocytes demonstrated a division of labor carried out by these two components in the immunoprotection developed by iNOS^−/−^ mice. While upregulated TNF-α secretion avoided precocious mortality and organized pulmonary lesions, the increased expansion of CD8^+^ T cells controlled fungal growth and secretion of pro-inflammatory cytokines. Both, TNF-α and CD8^+^ T cells were involved in the enhanced recruitment of inflammatory cells to the lungs. This protective effect was persistent, but excessive inflammatory reactions were possibly controlled by the increased expansion of Treg cells at late stages of immunity developed by NOS^−/−^ mice. Altogether, these mechanisms appear to confer sustained protection to iNOS^−/−^ mice, which, despite the elevated fungal loads, presented increased survival time and better disease outcome.

In our model, we could verify that iNOS deficiency seems to be compensated by the deviation of the immune response to a more pronounced Th1 pattern. The lower fungal loads were concomitant with the high levels of pulmonary TNF-α produced by *P.brasiliensis* infected NO-deficient mice at the 2^nd^ week of infection. Interestingly, it has been reported that low concentrations of NO enhanced Th1 immunity by increasing the expression of IL-12 receptor in T cells although high NO concentrations are cytotoxic [44], [46]. Interestingly, alternative mechanisms for the immunosuppressive activity of NO production have recently been described. It was demonstrated that NO suppresses NALP3 inflammasome activation by nitrosylation of NALP3 proteins resulting in decreased synthesis of mature IL-1 beta and IL-18, impaired Th1 immunity and NK cell activation [47]. Considering this information, it is tempting to speculate that the enhanced Th1 immunity developed by iNOS-deficient mice could be associated with increased NALP3 inflammasome activity and enhanced IL-1/IL-18 production. Consistent with a prevalent Th1 pattern of immunity, by week 2, increased levels of IFN-γ-regulated isotypes (IgG2a and IgG3), were produced by iNOS^−/−^ mice (data not shown). Thus, these data are consistent with the benign forms of murine and human PCM, which are associated with prevalent type-1 immunity [21]. Allied with type-1 cytokine production, an increased number of activated lymphocytes and macrophages was found in the lungs of iNOS-deficient mice suggesting the development of enhanced DTH reactions at the site of infection.

iNOS-deficient alveolar macrophages showed fungal loads equivalent to those detected in lung tissue and did not present an increased fungal ability when activated by IFN-γ. Several investigations showed the fundamental role of NO in *P. brasiliensis* killing by IFN-γ activated macrophages [2], [23], although NO-independent mechanisms were reported to be associated with TNF-α-activated human and murine macrophages [48], [49]. Our studies with inflammatory macrophages confirmed that iNOS^−/−^ macrophages are refractory to IFN-γ and TNF-α activation. Furthermore, the reduced CFU numbers displayed by these cells could not be ascribed to increased IDO expression, because the CFU counts were not modified by 1MT treatment. Importantly, iNOS^−/−^ macrophages were also shown to have a decreased ability to adhere to and ingest yeast cells, which possibly explains the low CFU numbers detected both in the *in vitro* and *in vivo* infections. Absence of iNOS expression was also associated with an M2-like behavior of macrophages. Indeed, these cells expressed high levels of arginase-1 and TGF-β and were associated with low levels of IL-12. This anti-inflammatory behavior appears to explain why iNOS-deficient cells were not activated by IFN-γ and TNF-α and explains why the iNOS-deficient cells do not express elevated levels of IDO, an enzyme primarily induced by IFN-γ activation [45]. However, iNOS-deficient cells expressed high levels of TNF-α, which is potentially associated with an efficient activation of dendritic cells and increased migration of inflammatory cells to the site of infection.

Interestingly, the behavior of iNOS^−/−^ macrophages is similar to the behavior of macrophages and dendritic cells from mice (strain A/J) resistant to *P. brasiliensis* infection [27], [35]. A/J cells are poorly activated by IFN-γ and IL-12 and show an impaired NO production and killing ability. Concomitant with elevated production of TGF-β, A/J macrophages produce high levels of TNF-α that contribute to the late but consistent cellular immunity and immunoprotection developed by this mouse strain [27], [35], [50].

Our data have also demonstrated that in pulmonary PCM, early NO production inhibits the activation and migration of CD4^+^ and CD8^+^ T cells to the site of infection. This finding was not previously described in murine PCM, but was reported in other experimental models where less severe infections, mainly due to increased IFN-γ production and CD4^+^ Th1-skewed immune responses, were observed in iNOS^−/−^ mice [4], [44], [46], [51], [52]. The increased influx of T cells and macrophages was concomitant with elevated levels of pulmonary TNF-α, a proinflammatory cytokine that enhances the maturation of antigen presenting cells and induces increased expression of adhesion molecules on endothelial cells [53]–[55]. Thus, the enhanced secretion of TNF-α appeared to have amplified the afferent and the efferent phases of immunity, by increasing T cell sensitization and further migration to the site of *P. brasiliensis* infection.

Despite the less severe infection at the 2^nd^ week, iNOS^−/−^ mice were not able to sustain this behavior, and elevated fungal burdens were seen in their lungs at later periods. Despite the yet significantly increased presence of activated CD4^+^ T cells in iNOS^−/−^ mice, no differences in the number or activation of CD8^+^ T cells were detected. This decreased influx of effector CD8^+^ T cells was parallel to the decreased activation of macrophages, indicating, as we previously demonstrated, the important role of this T cell subset in the immunoprotection of pulmonary PCM [21], [56].

Our studies appear to indicate that at an early phase of infection NO does not affect the expansion of CD4^+^CD25^+^Foxp3^+^ regulatory T cells. However, late in infection (week 10), iNOS deficiency supported the expansion of this regulatory T cell subset. These findings suggest that absence of NO production led to an early enhanced T cell immunity, but the excessive lung inflammation was lately avoided by increased expansion of Treg cells. The M2-like profile of inflammatory macrophages appears to have contributed to the expansion of Treg cells and the controlled tissue pathology observed in iNOS^−/−^ mice. This Treg-associated mechanism controlling effector immunity was not previously described for the NO-induced immunosuppression in murine PCM, and can be added to the regulatory mechanisms mediated by unbalanced NO production. Moreover, our data on the presence of CD4^+^CD25^+^Foxp3^+^ Treg cells at the site of infection are in good agreement with ours [57]–[59], and others [60], [61] reports showing a late enhancement of Foxp3^+^ Treg cells associated with increased immunological responses and pathogen burden.

Interestingly, the histopathological examination of lungs was consistent with the important role of TNF-α in the organization of granulomatous lesions [62], [64]. Indeed, the high influx of inflammatory T cells and macrophages into the lungs of iNOS^−/−^ mice, associated with the locally increased levels of TNF-α resulted in more organized lesions at week 10, which appeared to have overridden the elevated fungal loads due to the lack of NO synthesis. Furthermore, this pattern of lesion organization appeared to be able to restrain fungal dissemination to distant organs, since despite the lately increased pulmonary fungal burdens, no differences were detected in the liver and spleen of iNOS^−/−^ mice. The increased survival time of iNOS-deficient mice appears to underline the possible protective effect of well-organized lesions.

It is known that arginine catabolism is mediated by two types of enzymes: the nitric oxide synthases convert arginine to citrulline and NO, while the arginases hydrolyze arginine to urea and ornithine. The latter component is necessary for the production of proline and is essential for the synthesis of collagen, which is the main component of the extracellular matrix (ECM) observed in granulomatous lesions [65]. As here shown, iNOS-deficient macrophages only express arginase-1, which may have contributed to the expression of ECM components and the compact organization of granulomas observed in iNOS-deficient mice. It is also known that NO has a modulatory effect on the expression and activity of zinc-dependent metalloproteases (MMPs), which decrease the deposition and accumulation of extracellular matrix (ECM) proteins [66]. Some studies have demonstrated that NO induces the activation of MMP2 and MMP9, which are two enzymes that have an anti-fibrotic effects through the degradation of ECM proteins (laminin, collagen, elastin, etc.) and pro-cytokines involved in fibroblast activation and granuloma organization (e.g., pro-TNF-α and pro- TGF-β) [66], [67], [68]. In the intraperitoneal model of PCM using WT and iNOS^−/−^ mice [69], NO production was associated with increased MMP9 activity and the loose organization of granulomas developed by WT mice. In the pulmonary model employed in the present study we suppose that the NO produced by the WT mice inhibited the production of TNF-α, which is required for granuloma organization, and of TGF-β, which is needed for tissue repair and Treg cell expansion. Consistent with this possibility, infected iNOS^−/−^ macrophages expressed elevated levels of TGF-β (Fig. 5), which is a cytokine involved in fibroblast activation, enhanced synthesis of ECM, and Treg cell differentiation. The TGF-β-induced Treg cell differentiation apparently contributed to the controlled tissue pathology even in the presence of the augmented immune response of the iNOS^−/−^ mice. The study by Nishikako et al. [69] revealed additional important information on the influence of NO in granuloma formation and disease outcome. At late stages of infection (120 days post-infection, not evaluated in our model), i.p. infected iNOS^−/−^ mice presented decreased fungal loads in their well-organized granulomas, which demonstrated the efficiency of their inflammatory reactions. Furthermore, as observed here, iNOS^−/−^ mice showed decreased mortality rates when compared with WT mice.

In vivo depletion of TNF-α abrogated important advantages conferred by NO deficiency since only the iNOS^−/−^ strain showed precocious mortality rates associated with non-organized pulmonary lesions. Importantly, early after interrupting anti-TNF treatment, the levels of TNF-α returned to normal levels and a massive influx of activated T cells and macrophages into the lungs occurred in both mouse strains. Therefore, it became clear that besides mortality rates and organization of lesions, TNF-α also controlled the migration of inflammatory cells to the site of infection. As a whole, these results led us to demonstrate that the concomitant deficiency of NO and TNF-α is fatal to iNOS-deficient mice, whereas the still preserved ability of NO synthesis by TNF-depleted WT mice appeared to rescue this mouse strain from precocious mortality.

Depletion experiments of CD8^+^ T cells revealed the important role of this T cell subset in the early immunoprotection of iNOS^−/−^ mice. Thus, the early differences in fungal loads were abrogated, and the influx of inflammatory cells was markedly impaired only in CD8-depleted mice iNOS^−/−^ mice. Studies on the phenotype of DCs at the site of infection showed that anti-CD8 treatment did not alter the presence of CD8^+^ lymphoid DCs, suggesting that CD8^+^ T lymphocytes, and not lymphoid DCs, played an important control of fungal growth and inflammatory reactions mediated by T cells and macrophages. We could verify by intracellular cytokine staining that in iNOS^−/−^ mice, the depletion of CD8^+^ T cells resulted in decreased numbers of CD4^+^ (IFN-γ) and CD8^+^ (IFN-γ and TNF-α) T cells, supporting the proinflammatory feature of this T cell subpopulation. This fact was consistent with the diminished influx of inflammatory cells observed in the lungs of CD8-depleted iNOS^−/−^ mice. In WT mice, however, the depletion of CD8^+^ cells had a negligible effect, and this appears to reflect the concomitant increase of IFN-γ and TNF-α CD4^+^ T cells with decreased numbers of CD8^+^ T cells secreting the same proinflammatory cytokines in the lungs. The increased presence of IL-4^+^ CD4^+^ T cells appeared to have exerted a negligible effect in the inflammation mounted by WT mice.

In conclusion, this work brought new information regarding the role of NO synthesis in experimental PCM. We demonstrated the protective effect of iNOS deficiency in pulmonary PCM. This protective effect appeared to be mediated by increased type-1 inflammatory reactions regulated by TNF-α production and expansion of IFN-γ and TNF-α-producing T cells.
